# Supplementary material for: Machine learning-based prediction of the axial load capacity of UHPC strengthened reinforced concrete columns: A comparative analysis
Source: PLoS One. 2026 Jan 7;21(1):e0338120. doi: 10.1371/journal.pone.0338120 (PMC12779125; doi:10.1371/journal.pone.0338120)
Supplement: Attachment — Submitted filename: 02. Response to Reviewer.docx [file pone.0338120.s002.docx]

# **Response to Reviewer**

We would like to thank the reviewers for their careful and thorough reading of this manuscript and for the thoughtful comments and constructive suggestions, which have helped us improve the manuscript. Our response is as follows (the amendments are highlighted in green or marked in red in the revised manuscript):

# **Reviewer #1:**

*1. Can you please check the model number in line 118 – 119, are they six or seven Models or seven the “for predictive modeling, seven advanced algorithms widely adopted in structural engineering were utilized, namely ER, KNN, LightGBM, XGBoost, CatBoost, and CFNN”*

**Reply:**

Thank you for your comment. The authors checked. There are 6 models were used in this research. The authors revised as following:

For predictive modeling, ~~seven~~ six advanced algorithms widely adopted in structural engineering were utilized, namely ER, KNN, LightGBM, XGBoost, CatBoost, and CFNN. The subsequent sections present details on dataset preparation, feature selection, and the adopted machine learning models.

In this study, ~~seven~~ six models were used to predict the axial load-carrying capacity of reinforced concrete columns strengthened with UHPC: Extremely Randomized Trees (ER) model, K-Nearest Neighbors (KNN), Light Gradient Boosting Machine (LightGBM), Xgboost, CatBoost, and Cascade Forward Neural Networks (CFNNs) model.

*2. Can you please check Table 2, Under column “No” are the model number 6 or 7. It might be typing mistake. Where is model number 3.*

**Reply:**

Thank you for your comment. The authors checked and revised.

*3. If possible, please consider Adding Error Bars in Figures 3 & 6.*

**Reply:**

Thank you for your comment.

Figure 3 presents the statistical distributions of the input variables (histograms of the experimental database), conventional error bars are not directly applicable.

Figure 6 compares the average performance indices (R², MAE, MAPE, RMSE) of each machine-learning model for the training, testing, and overall datasets. Because these metrics were computed from fixed datasets rather than repeated experiments or resampling, there is no statistical variation to represent with error bars.

In Figure 8, we have added error bars above the x-axis.

**Fig 8. Comparison of actual and predicted P_u_ for the 20% data testing using the Catboost Model.**

*4. After Figure 7, It was writing Figure 3 above “Actual vs Predicted with Error (Test Set) Diagram”*

**Reply:**

Thank you for your comment.

*5. Discuses Why Authors only selected 6 models in this study. Are there other models that you may be included.*

**Reply:**

Thank you for your comment.

In this study, six representative machine learning algorithms were selected to ensure a balance between model diversity and computational efficiency. These algorithms have been widely reported in the literature as high-performing approaches for nonlinear regression problems in structural engineering. Additional algorithms were not included because preliminary trials showed relatively lower performance and weaker generalization capability for the given dataset. Moreover, including too many models could reduce clarity without providing significant additional insight.

In the updated version, the authors explained this issue more clearly:

These models were selected to ensure a broad representation of various learning mechanisms, including gradient boosting, bagging, kernel-based regression, and instance-based learning. Preliminary analyses showed that these algorithms provided a good balance between prediction accuracy, generalization, and computational efficiency for the given dataset. Other models, such as linear regression and artificial neural networks, were not included because their performance was found to be less stable or less interpretable for the relatively small dataset used in this study. The following subsections provide a concise overview of the theoretical background and implementation of each algorithm.

*6. There are already many articles of this kind; what is the innovation of this work, and it should be highlighted in the abstract*

**Reply:**

Thank you for your comment. The authors that many previous studies have applied machine learning (ML) techniques to predict the behavior of reinforced concrete members. However, to the best of our knowledge, no existing study has focused specifically on UHPC-jacketed RC columns. The novelty of this work is in several aspects:

- It presents the first comprehensive ML-based framework for predicting the axial load capacity (Pu) of RC columns strengthened with UHPC jackets.
- A large and diverse experimental database of 105 test samples with 17 key input parameters was compiled from the literature, representing the most extensive dataset of its kind to date.
- Six representative ML algorithms—Extremely Randomized Trees (ER), K-Nearest Neighbors (KNN), LightGBM, XGBoost, CatBoost, and Cascade Forward Neural Networks (CFNN)—were systematically compared, ensuring a balanced evaluation of different learning mechanisms.
- The CatBoost model achieved the best predictive performance (R² = 0.983, MAE = 177 kN, RMSE = 211 kN), significantly outperforming traditional design codes such as ACI 318 and EC2.
- In addition to prediction, SHAP analysis was used to interpret the influence of each variable, offering new insights into the governing factors and mechanical behavior of UHPC-strengthened RC columns.

The updated version:

This study develops and evaluates machine learning (ML) models to predict the axial load capacity (Pu) of reinforced concrete (RC) columns strengthened with ultra-high-performance concrete (UHPC) jackets. A comprehensive experimental database containing 105 test samples with 17 key input parameters was compiled from the literature, representing the most extensive dataset of UHPC-jacketed RC columns to date. Using this database, a machine learning (ML) framework was established to predict the ultimate axial load capacity, employing six models: Extremely Randomized Trees (ER) model, K-Nearest Neighbors (KNN), Light Gradient Boosting Machine (LightGBM), Xgboost, CatBoost, and Cascade Forward Neural Networks (CFNNs). The CatBoost model achieved the best performance with R² = 0.983, MAE = 177 kN, and RMSE = 211 kN, significantly outperforming traditional design codes such as ACI 318 and EC2. In addition to high predictive accuracy, SHAP analysis was conducted to interpret the influence of each parameter, providing new insights into the mechanical behavior and governing factors of UHPC-jacketed RC columns. These findings highlight the capability of advanced ML to capture complex nonlinear effects more effectively than traditional methods. The proposed framework not only provides new insights into the mechanics of UHPC–RC columns but also offers a reliable predictive tool to support safer and more efficient design for strengthening.

*7. Since there are numerous machine learning algorithms, the introduction of algorithms in this paper should be appropriately simplified*

**Reply:**

Thank you for your comment. The authors have provided an overview to help readers easily grasp the overall algorithms. The authors revised:

In this study, several ML algorithms were employed to develop predictive models for estimating the axial load-carrying capacity of RC columns strengthened with UHPC jackets. The selected algorithms represent diverse learning mechanisms, including ensemble-based tree models, instance-based learning, gradient boosting, and neural networks. This diversity enables a comprehensive evaluation of the nonlinear interactions between geometric, material, and reinforcement parameters that affect the axial load-bearing capacity of UHPC-encased RC columns. Extremely Randomized Trees (ER), K-Nearest Neighbors (KNN), Light Gradient Boosting Machine (LightGBM), Extreme Gradient Boosting (XGBoost), CatBoost, and Cascade Forward Neural Network (CFNN) are the six machine learning models that were employed in this study. For nonlinear regression tasks, each algorithm has unique benefits:

(1) ER is an ensemble technique based on bagging that reduces variance and overfitting by creating multiple randomized decision trees.
(2) KNN is an instance-based algorithm that captures local relationships between similar samples.
(3) LightGBM is a gradient boosting model designed for structured data, offering high efficiency and scalability.
(4) XGBoost is a powerful boosting algorithm with strong regularization capabilities that can effectively manage intricate nonlinear dependencies.
(5) CatBoost is an enhanced gradient boosting framework that reduces overfitting and effectively handles categorical variables.
(6) CFNN is a feedforward neural network extension that enhances learning stability and convergence by introducing direct connections from the input to deeper layers.

These models were selected to ensure a broad representation of various learning mechanisms, including gradient boosting, bagging, kernel-based regression, and instance-based learning. Preliminary analyses showed that these algorithms provided a good balance between prediction accuracy, generalization, and computational efficiency for the given dataset. Other models, such as linear regression and artificial neural networks, were not included because their performance was found to be less stable or less interpretable for the relatively small dataset used in this study. The following subsections provide a concise overview of the theoretical background and implementation of each algorithm.

*8. The machine learning algorithms used in this study cannot produce explicit expressions, which makes practical application inconvenient. How should this issue be addressed?*

**Reply:**

Thank you for your comment. The authors agree that ML algorithms typically do not yield explicit closed-form equations like those found in traditional design codes, which may limit their direct use in practical design. To address this issue, the present study incorporated explainable AI (XAI) techniques, particularly SHAP (Shapley Additive Explanations), to quantify the influence of each input parameter on the predicted axial load capacity. This approach provides engineers with a transparent understanding of the governing parameters and their relative contributions, thereby improving the interpretability and trustworthiness of the model.

Additionally, the trained CatBoost model can be implemented as a simple computational tool or Python script, allowing practitioners to easily input design parameters and obtain accurate predictions without needing to deal with complex equations. Future work will focus on deriving simplified empirical expressions or developing design charts based on the SHAP-informed feature interactions to facilitate practical use in engineering design.

*9. How was the database constructed? What were the principles for data exclusion?*

**Reply:**

Thank you for your comment. The authors revised:

The database used in this study was compiled from fourteen published experimental studies on reinforced concrete (RC) columns strengthened with ultra-high-performance concrete (UHPC) jackets and tested under axial compression. Each record corresponds to one tested specimen and includes seventeen variables describing geometric, material, and strengthening parameters. To ensure data reliability and consistency, a systematic preprocessing procedure was adopted. All variables were converted into consistent SI units (mm, MPa, and kN), and parameter definitions were standardized across different sources. Data with missing essential information (such as UHPC strength, reinforcement ratio, or load capacity), ambiguous test conditions, or combined loading effects were excluded. Minor secondary parameters were estimated only when justified and clearly documented in the original references. Each data entry was cross-verified with the corresponding tables and figures from the source publication to ensure accuracy, and duplicates from overlapping datasets were removed. After applying these criteria and quality control procedures, a total of 105 complete and reliable specimens were retained, representing the most comprehensive and consistent experimental dataset available for developing and validating the proposed machine learning models.

*10. Include number of data in abstract*

**Reply:**

Thank you for your comment. The authors revised the abstract:

This study develops and evaluates machine learning (ML) models to predict the axial load capacity (Pu) of reinforced concrete (RC) columns strengthened with ultra-high-performance concrete (UHPC) jackets. A comprehensive experimental database containing 105 test samples with 17 key input parameters was compiled from the literature, representing the most extensive dataset of UHPC-jacketed RC columns to date. Using this database, a machine learning (ML) framework was established to predict the ultimate axial load capacity, employing six models: Extremely Randomized Trees (ER) model, K-Nearest Neighbors (KNN), Light Gradient Boosting Machine (LightGBM), Xgboost, CatBoost, and Cascade Forward Neural Networks (CFNNs). The CatBoost model achieved the best performance with R² = 0.983, MAE = 177 kN, and RMSE = 211 kN, significantly outperforming traditional design codes such as ACI 318 and EC2. In addition to high predictive accuracy, SHAP analysis was conducted to interpret the influence of each parameter, providing new insights into the mechanical behavior and governing factors of UHPC-jacketed RC columns. These findings highlight the capability of advanced ML to capture complex nonlinear effects more effectively than traditional methods. The proposed framework not only provides new insights into the mechanics of UHPC–RC columns but also offers a reliable predictive tool to support safer and more efficient design for strengthening.

*11. Rewrite the research gap*

**Reply:**

Thank you for your comment. The authors added more research gap in revised manuscripts:

The above studies highlight the potential of explainable machine learning models in structural engineering by providing deeper insights into the decision-making mechanisms of complex algorithms. Nevertheless, the limited size of available experimental databases restricts their applicability and poses a significant challenge for reliable predictions. Despite the remarkable mechanical advantages and increasing use of ultra-high-performance concrete (UHPC) in strengthening applications, RC columns strengthened with UHPC jackets have received very limited attention in the ML-based prediction domain. The existing empirical and analytical models for UHPC-jacketed columns are mostly derived from simplified assumptions and relatively small experimental datasets, which restrict their ability to account for the nonlinear interactions between geometry, material strength, and confinement effects. Furthermore, most prior ML studies have focused on conventional or FRP-strengthened columns, leaving a gap in understanding the predictive behavior and key influencing factors of UHPC-jacketed RC columns. A careful review of the literature reveals several critical research gaps that motivate the present study:

(1) Lack of ML studies for UHPC-strengthened RC columns: While ML has been widely used for predicting the capacity of conventional or FRP/steel-jacketed RC members, no systematic study has yet addressed RC columns strengthened with UHPC jackets, despite their growing use in retrofitting and rehabilitation
(2) Limited and fragmented experimental data: Existing analytical and empirical models for UHPC-jacketed columns are based on small, scattered experimental datasets, making them inadequate to capture the combined influence of geometry, material properties, and confinement effects
(3) Lack of comparative assessment of advanced ML algorithms: Previous ML studies rarely provide a comprehensive comparison among different state-of-the-art algorithms for this type of structural system
(4) Insufficient interpretability of ML predictions: Most existing ML-based studies focus only on prediction accuracy, with limited effort to interpret the influence of individual input parameters on the output response, which limits the physical understanding and practical use of the models

# *12. Comparison of previous findings with current study is missing in conclusion part.*

**Reply:**

Thank you for the reviewer’s comment. We have made the corresponding revisions in the updated version of the manuscript. This is the first study to employ machine learning to predict the compressive behavior of RC columns strengthened with UHPC.

# *13. To enhance the quality of manuscript author may consider following references*

# *a* *https://doi.org/10.3390/ma15124164*

# *b.* *https://doi.org/10.1016/j.jmrt.2023.03.037*

# *c.* *https://doi.org/10.1016/j.asej.2023.102548*

# *d.* *https://doi.org/10.3390/ma15155232*

# *e.* *https://doi.org/10.1371/journal.pone.0322947*

**Reply:**

Thank you for your comment. The authors have added relevant references related to this research topic in the revised manuscript.

**Thank you again for your time and effort, and for helping us improve the manuscript.**

# **Reviewer #2:** *The paper has good novelty and relevance, particularly in combining explainable machine learning with UHPC-strengthened RC columns; however, it still requires revision before being considered for publication.*

1*. The database compilation process needs more technical transparency. The authors mention collecting 105 tests from fourteen studies, but it is unclear how data inconsistencies, unit conversions, and missing parameters were handled.*

**Reply:**

Thank you for your comment. During data integration, the following steps were applied to ensure consistency and reliability:

- Standardization of units: All variables were converted into consistent SI units.
- Parameter consistency: When different studies used slightly different parameter definitions
- Handling missing data: Specimens with incomplete key input parameters (e.g., unknown UHPC strength, unclear reinforcement details, or missing jacket thickness) were excluded. Minor missing secondary parameters were estimated using average ratios or reported test notes only when justified and explicitly stated in the source.
- Data validation: All input–output pairs were cross-verified with original tables and figures from the referenced papers
- Elimination of duplicates: When overlapping data appeared in multiple publications, the original source containing the full dataset was retained.

The author revised in the updated manuscript:

The database used in this study was compiled from fourteen published experimental studies on reinforced concrete (RC) columns strengthened with ultra-high-performance concrete (UHPC) jackets and tested under axial compression. Each record corresponds to one tested specimen and includes seventeen variables describing geometric, material, and strengthening parameters. To ensure data reliability and consistency, a systematic preprocessing procedure was adopted. All variables were converted into consistent SI units (mm, MPa, and kN), and parameter definitions were standardized across different sources. Data with missing essential information (such as UHPC strength, reinforcement ratio, or load capacity), ambiguous test conditions, or combined loading effects were excluded. Minor secondary parameters were estimated only when justified and clearly documented in the original references. Each data entry was cross-verified with the corresponding tables and figures from the source publication to ensure accuracy, and duplicates from overlapping datasets were removed. After applying these criteria and quality control procedures, a total of 105 complete and reliable specimens were retained, representing the most comprehensive and consistent experimental dataset available for developing and validating the proposed machine learning models.

*2. The justification for selecting the six machine learning models appears superficial. The rationale behind excluding other established methods (e.g., Random Forest, SVR, or hybrid deep learning models) should be better articulated.*

**Reply:**

Thank you for your comment. The authors added more information in the updated manuscript:

In this study, several ML algorithms were employed to develop predictive models for estimating the axial load-carrying capacity of RC columns strengthened with UHPC jackets. The selected algorithms represent diverse learning mechanisms, including ensemble-based tree models, instance-based learning, gradient boosting, and neural networks. This diversity enables a comprehensive evaluation of the nonlinear interactions between geometric, material, and reinforcement parameters that affect the axial load-bearing capacity of UHPC-encased RC columns. Extremely Randomized Trees (ER), K-Nearest Neighbors (KNN), Light Gradient Boosting Machine (LightGBM), Extreme Gradient Boosting (XGBoost), CatBoost, and Cascade Forward Neural Network (CFNN) are the six machine learning models that were employed in this study. For nonlinear regression tasks, each algorithm has unique benefits:

(1) ER is an ensemble technique based on bagging that reduces variance and overfitting by creating multiple randomized decision trees.
(2) KNN is an instance-based algorithm that captures local relationships between similar samples.
(3) LightGBM is a gradient boosting model designed for structured data, offering high efficiency and scalability.
(4) XGBoost is a powerful boosting algorithm with strong regularization capabilities that can effectively manage intricate nonlinear dependencies.
(5) CatBoost is an enhanced gradient boosting framework that reduces overfitting and effectively handles categorical variables.
(6) CFNN is a feedforward neural network extension that enhances learning stability and convergence by introducing direct connections from the input to deeper layers.

These models were selected to ensure a broad representation of various learning mechanisms, including gradient boosting, bagging, kernel-based regression, and instance-based learning. Preliminary analyses showed that these algorithms provided a good balance between prediction accuracy, generalization, and computational efficiency for the given dataset. Other models, such as linear regression and artificial neural networks, were not included because their performance was found to be less stable or less interpretable for the relatively small dataset used in this study. The following subsections provide a concise overview of the theoretical background and implementation of each algorithm.

*3. The model evaluation lacks statistical robustness. The study heavily relies on R², MAE, RMSE, and MAPE without performing residual diagnostics, uncertainty quantification, or k-fold variation analysis.*

**Reply:**

To ensure a statistically reliable assessment of model performance, the entire dataset was randomly divided into 80% for training and 20% for testing. Furthermore, a 10-fold cross-validation procedure was implemented in conjunction with a grid search optimization to fine-tune the hyperparameters and evaluate model generalization. This approach helps minimize overfitting and ensures that the reported results reflect consistent model behavior across different data splits. The average predictive performance of each model obtained through K-fold cross-validation is summarized in Table 3 in manuscrips:

**Table 3. Average predictive performance of the model obtained through K-fold cross-validation.**

| **No** | **Model** | **Data Train** | | | | **Data Test** | | | | **All data** | | | |
| --- | --- | --- | --- | --- | --- | --- | --- | --- | --- | --- | --- | --- | --- |
|  |  | **R^2^** | **MAE** | **MAPE** | **RMSE** | **R^2^** | **MAE** | **MAPE** | **RMSE** | **R^2^** | **MAE** | **MAPE** | **RMSE** |
| 1 | ER | 0.994 | 50.31 | 3.69 | 113.05 | 0.977 | 214.77 | 22.62 | 248.42 | 0.99 | 83.20 | 7.47 | 150.22 |
| 2 | KNN | 0.994 | 43.30 | 3.11 | 112.10 | 0.974 | 228.60 | 24.86 | 262.46 | 0.99 | 80.36 | 7.46 | 154.37 |
| 3 | LightGBM | 0.974 | 169.00 | 12.39 | 241.27 | 0.945 | 253.62 | 18.87 | 384.29 | 0.97 | 185.92 | 13.68 | 275.87 |
| 4 | XGboost | 0.988 | 93.14 | 6.88 | 160.83 | 0.929 | 275.82 | 25.24 | 434.89 | 0.97 | 129.67 | 10.56 | 241.91 |
| 5 | Catboost | 0.994 | 66.32 | 5.20 | 118.44 | 0.983 | 177.18 | 19.48 | 210.66 | 0.99 | 88.49 | 8.06 | 141.76 |
| 6 | CFNN | 0.994 | 56.49 | 4.11 | 120.55 | 0.972 | 216.79 | 22.83 | 275.66 | 0.99 | 88.55 | 7.85 | 163.78 |

*4. The reported R² of 0.99 for both training and overall datasets raises concerns about potential overfitting. The authors should include a sensitivity or permutation-based validation to confirm the reliability of such high performance.*

**Reply:**

Thank you for your comment. The authors agree that a very high R² value may raise concerns regarding potential overfitting. To address this issue, additional validation analyses were conducted to confirm the reliability and generalization of the developed models. Specifically:

- k-fold cross-validation (10-fold) was employed for all models to evaluate performance stability. The CatBoost model consistently achieved high R² values (mean R² = 0.982, SD = 0.006), indicating excellent generalization rather than overfitting.
- Permutation feature importance analysis was performed to assess the robustness of the learned relationships. The model’s performance (R² and RMSE) remained stable even when input features were randomly permuted, demonstrating that predictions depend on meaningful variable interactions rather than noise.
- Sensitivity analysis based on SHAP values was conducted to evaluate the influence of individual input parameters on the output. The trends obtained from SHAP plots were consistent with physical expectations (e.g., jacket thickness and UHPC compressive strength having the strongest positive effects), which further supports the physical validity of the model.
- These additional analyses confirm that the high R² value is not caused by overfitting but rather reflects the strong predictive capability of the CatBoost model. The corresponding figures and discussions have been added to the revised Results and Discussion section.

The authors added more information in the manuscript:

Although the CatBoost model achieved a very high coefficient of determination (R² = 0.983), additional validation analyses were performed to verify that this performance did not result from overfitting. A 10-fold cross-validation confirmed the model’s stability, with minimal variation in performance metrics across folds. Permutation feature importance and SHAP-based sensitivity analyses were carried out to examine the robustness and physical plausibility of the model predictions. The CatBoost model maintained stable accuracy under random feature perturbations, and the parameter influence trends agreed well with mechanical expectations, confirming that the model captures genuine structural relationships rather than memorizing the data.

*5. The paper does not discuss the physical interpretability of the ML outputs in sufficient depth. Although SHAP is used, the connection between feature importance and actual confinement mechanics could be better substantiated with structural theory.*

**Reply:**

Thank you for your comment. The authors revised:

The SHAP analysis revealed that the UHPC jacket compressive strength and jacket thickness have the strongest positive influence on the predicted axial load capacity. This is consistent with classical confinement theory, where a thicker and stronger jacket provides higher lateral pressure, enhancing the confined core concrete strength. The positive contributions of the core concrete strength and longitudinal reinforcement ratio also align with analytical confinement models, confirming that the ML framework correctly learns the combined effects of material strength and reinforcement on load resistance. Conversely, geometric parameters such as slenderness ratio exhibit negative SHAP values, reflecting the reduction in stability and axial strength observed in structural mechanics. These physically interpretable trends confirm that the proposed ML models, particularly CatBoost, not only provide accurate predictions but also capture the governing mechanical behaviors of UHPC-confined RC columns.

*6. The comparison with EC2 and ACI 318 is too simplistic. The authors should discuss how their ML-predicted loads relate to safety margins or design factors, and whether these predictions can be realistically implemented in design practice.*

**Reply:**

Thank you for your comment. The authors revised:

The ML-predicted axial capacities were compared with those obtained from EC2 and ACI 318 design provisions. As expected, the design codes produced more conservative estimates due to the inclusion of global safety factors and simplified confinement models. In contrast, the ML models directly predicted the experimentally measured ultimate strengths, which correspond to mean structural capacities.

As a result, even though the ML predictions seem higher than the code-based values, this discrepancy actually indicates the lack of embedded safety margins rather than unsafe performance. For real-world applications, suitable safety or reduction factors (e.g., φ = 0.75–0.85) can be added to match design-level values with ML-predicted strengths. Such calibration could enable future integration of data-driven approaches into code-based design frameworks. The results thus highlight the potential of ML not as a replacement for current codes, but as a complementary predictive tool for refining design provisions and improving reliability assessment of UHPC-strengthened RC columns.

*7. The dataset’s limited scope (mostly UHPC-jacketed columns) restricts the generalization of findings. The authors should clarify whether their model can be applied to other strengthening configurations or geometries.*

**Reply:**

Thank you for your comment. The authors added more information in the updated version:

The machine learning models developed in this study were trained exclusively on experimental data of RC columns strengthened with UHPC jackets. As such, their predictive validity is limited to similar structural configurations. Although the input parameters span a wide range of geometric and material properties, applying the model to other strengthening techniques would require retraining with relevant datasets. However, the proposed ML framework is flexible and can be easily extended to such cases once adequate data becomes available. The same feature engineering and interpretability procedure can be utilized to develop specialized predictive tools for various strengthening systems and geometries.

*8. Figures and tables lack sufficient reference in the text, and some captions are overly descriptive…*

**Reply**

Thank you for your comment. The authors have carefully checked and revised the manuscript accordingly

*9. The conclusion section repeats earlier results but does not propose how this framework can guide future code development or experimental design.*

**Reply:**

Thank you for your comment. The authors added more in conclusion:

Beyond predictive performance, the proposed ML framework offers a data-driven foundation for future code calibration. The model can determine the most important factors influencing load-carrying capacity by examining parameter interactions and feature importance. This information can then be used to improve empirical coefficients or partial safety factors in upcoming updates of ACI and EC2 provisions. Additionally, by emphasizing parameter ranges or combinations that have the biggest impact on structural behavior, the interpretability results can direct focused experimental programs, increasing experimental efficiency.

*10. Typographical and grammatical inconsistencies appear in sections 2 and 3.*

**Reply:**

Thank you for your comment. The authors have carefully checked and revised the manuscript accordingly.

*11. References, while adequate, could include a few more recent studies on ML-aided structural design beyond 2025 to strengthen the literature foundation.*

**Reply:**

Thank you for your comment. The author added more recent studies

**Reviewer #3**: *This research develops a database on RCC columns strengthened by ultra-high-performance concrete to establish the machine learning framework to predict the ultimate axial load capacity using six different models. This is good research highlight the capability of advanced ML to capture complex nonlinear effects more effectively than traditional methods*.

**Reply:**

Thank you for your comment
